# Nutritional, Chemical, Antioxidant and Antibacterial Screening of *Astragalus cicer* L. and *Astragalus glycyphyllos* L. Different Morphological Parts

**DOI:** 10.3390/foods14020250

**Published:** 2025-01-14

**Authors:** Saba Shahrivari-Baviloliaei, Agnieszka Konopacka, Liege Aguiar Pascoalino, Filipa Reis, Dawid Kunkowski, Spyridon A. Petropoulos, Pawel Konieczynski, Ilkay Erdogan Orhan, Alina Plenis, Agnieszka Viapiana

**Affiliations:** 1 Department of Analytical Chemistry, Faculty of Pharmacy, Medical University of Gdansk, 80-210 Gdansk, Poland; saba.shahrivari@gumed.edu.pl (S.S.-B.); dawid240200@gumed.edu.pl (D.K.); pawel.konieczynski@gumed.edu.pl (P.K.); 2 Department of Pharmaceutical Microbiology, Faculty of Pharmacy, Medical University of Gdansk, 80-210 Gdansk, Poland; agnieszka.konopacka@gumed.edu.pl; 3 Centro de Investigação de Montanha (CIMO), Instituto Politécnico de Bragança, Campus de Santa Apolónia, 5300-253 Bragança, Portugal; liegeaguiar@ipb.pt (L.A.P.); freis@ipb.pt (F.R.); 4 Laboratório Associado para a Sustentabilidade e Tecnologia em Regiões de Montanha (SusTEC), Instituto Politécnico de Bragança, Campus de Santa Apolónia, 5300-253 Bragança, Portugal; 5 Department of Agriculture, Crop Production and Rural Environment, University of Thessaly, Fytokou Street, 38446 Volos, Greece; petropoulos@uth.gr; 6 Department of Pharmacognosy, Faculty of Pharmacy, Gazi University, Ankara 06330, Türkiye; ilkay.erdoganorhan@lokmanhekim.edu.tr; 7 Department of Pharmacognosy, Faculty of Pharmacy, Lokman Hekim University, Ankara 06510, Türkiye

**Keywords:** *Astragalus*, bioactive properties, phytochemical composition, plant extract

## Abstract

The chemical composition and biological activity of *A. glycyphylos* and *A. cicer* are scarcely investigated. In this study, the nutritional and chemical profiles of *A. cicer* and *A. glycyphyllos*, considering their different morphological parts (leaves, fruits and roots), were assessed together with their antioxidant and antibacterial potential. Our results showed that carbohydrates are the major macronutrients in both *Astragalus* species (above 62 g/100 g dry weight—DW). High amounts of ash (above 4.6 g/100 g DW) and protein (above 13.0 g/100 g DW) were also identified, particularly in leaves and fruits of *A. cicer* and *A. glycyphyllos*. Moreover, *A. cicer* was richer in sugars than *A. glycyphyllos*, while roots of both *Astragalus* species were the richest of fatty acids. Ten phenolic compounds were identified, with gallic acid and quercetin being predominant, above 49.84 and 37.27 μg/g DW, respectively. The mineral analysis revealed zinc and iron as the major constituents. Regarding the plants’ antioxidant and antibacterial activity, both *Astragalus* species had antioxidant potential, and their water extracts showed antibacterial activity against *S. aureus* and *E. coli*. Altogether, these results provide insight into the potential of *A. glycyphyllos* and *A. cicer* as a source of nutritional benefits and active phytochemicals for many people, and they can be applied in the food sector as foods and as promising sources of natural ingredients.

## 1. Introduction

*Astragalus* L. is a genus of plants belonging to the Fabaceae family, consisting of 2000–3000 species and more than 250 taxonomic groups worldwide [1]. Moreover, *Astragalus* is considered to be one of the most diverse genera in the Fabaceae family [2] and is commonly used for feed and by wild animals as well as in food and medicinal formulations. Some species of *Astragalus* in Asia belong to the important natural source of gum tragacanth [3]. Furthermore, *Astragalus* species have been applied in medicine for about 2000 years. In Iranian traditional medicine, the crude herbal remedies produced from the roots of *Astragalus* species are used to treat a variety of ailments, including respiratory infections, diabetes, and leukemia [4]. The medicinal properties of *Astragalus* species are related to their chemical composition, while so far, more than 200 compounds have been discovered in several *Astragalus* species. According to modern chemical analysis, the major classes of compounds are polysaccharides, saponins, and flavonoids, which are the main biologically active constituents of *Astragalus* species [5]. *Astragalus* polysaccharides are used to treat muscle atrophy [6] and show the immunomodulatory effect. It makes them promising for the treatment of many diseases, including cancer, infection, type 1 diabetes, asthma, and autoimmune disease [7]. Moreover, recent research indicates that *Astragalus* polysaccharides and *Lactobacillus acidophilus* can better improve calcium absorption and osteoporosis [8,9]. Among *Astragalus* saponins, astragaloside IV is currently considered the most bioactive chemical component for its wide-ranging pharmacological effects, including anti-tumor [10], anti-inflammatory [11], anti-fibrosis [12], and immunomodulatory [13] actions in various diseases, such as cancer, inflammatory conditions, and fibrotic diseases. The active *Astragalus* flavonoid components are predominantly calycosin, formononetin and ononin and their antioxidant, antimicrobial, anti-inflammatory, immune-regulatory, and anti-tumor activities have been widely identified [14,15].

*Astragalus glycyphyllos* L. (liquorice milkvetch) and *Astragalus cicer* L. (cicer milkvetch) are perennial species, which are widespread throughout Europe and temperate Asia [16,17]. These species have been used in traditional medicine as well as for food purposes in several European countries [18,19]. *A. glycyphyllos* has been widely used in Bulgarian folk medicine as an antihypertensive, diuretic, and anti-inflammatory [20]. The herb can be administered as an infusion in cases of cardiac insufficiency, calculosis, or tachycardia [21]. In clinical trials, a 10% infusion of *A. glycyphyllos* displayed hypotensive, anticoagulant and diuretic activity [22,23]. Moreover, the phytochemical content of *A. glycyphyllos* suggests that it also possesses cytotoxic [23], neuroprotective [24], immunomodulatory [25], and antiviral activity [26], as well as antioxidant and hepatoprotective effects [27]. In the case of *A. cicer* in Belarusian folk medicine, the plant is used to treat heart and gastrointestinal diseases. Moreover, the plant has hypotensive, diuretic, bactericidal, and sedative effects [28].

Although *A. glycyphyllos* and *A. cicer* have been in use for a long time, the utilization of these *Astragalus* species is based only on empirical knowledge because there is a scarcity of data on their nutritional and chemical composition as well as on their bioactive properties, especially regarding their different morphological parts. Therefore, the aim of this study was to nutritionally and chemically characterize *A. cicer* and *A. glycyphyllos* water and hydromethanolic extracts prepared from different morphological parts (leaves, roots and fruits) and evaluate their antioxidant and antibacterial properties, supporting traditional uses of these species and, in the case of hydromethanolic extracts, as novel sources of bioactive compounds in food applications.

## 2. Materials and Methods

### 2.1. Samples

The plant samples were collected from the botanical garden of Maria Curie-Sklodowska University of Lublin (Poland) in September–October 2023, and they were taxonomically identified as *A. glycyphyllos* by Dr. Mykhaylo Chernetskyy and *A. cicer* by M. Sc. Anna Serewa. After being washed with water to remove dust, the plants were shade-dried for approximately 20 days at room temperature. Finally, the roots, fruits, and leaves of each plant species were separated and then pulverized in a water-cooled grinder (Knifetec 1095, Foss Tecator, Höganäs, Sweden) at 20 °C for 20 s.

### 2.2. Reagents, Standards and Instrumental

4-Chloro-7-nitrobenzofurazan (NBD-Cl), 2,2-diphenyl-1-picrylhydrazyl (DPPH reagent), and ten standards, including protocatechuic acid (PAT), gallic acid (GA), *p*-coumaric acid (pCA), vanillic acid (VA), cinnamic acid (CNA), ferulic acid (FA), quercetin (Q), rutin (RUT), naringenin (NAR) and apigenin (API), were purchased from Sigma-Aldrich (St. Louis, MO, USA) [29]. Other reagents were obtained from POCh (Gliwice, Poland). Redistilled water was prepared as already described by Saba Shahrivari-Baviloliaei et al. [29].

Tocopherols (α-, β-, γ-, and δ-isoforms) were purchased from Sigma (St. Louis, MO, USA). Racemic tocol, 50 mg/mL, was purchased from Matreya (State College, PA, USA). Methanol and hexane were of analytical grade and purchased from Fisher Scientific, Lisbon, Portugal.

### 2.3. Sample Preparation

Hydromethanolic and water extracts of the roots, fruits, and leaves of *A. glycyphyllos* and *A. cicer* were prepared according to the literature [30], with some modifications. For the hydromethanolic extracts, each plant sample (0.5 g) was sonicated for 30 min with 5 mL of the mixture of methanol and water (75:25, *v*/*v*) at 40 kHz and 150 W and temperature set at 30 °C (Emag, Salach, Germany) [30]. The suspension was centrifuged for 10 min at 7871 rcf (EBA-20S, Hettich, Tuttlingen, Germany) and transferred into a volumetric flask [30]. After repeating this procedure twice, the extracts were combined and, next, diluted up with the mixture of methanol and water (75:25, *v*/*v*) to 10 mL [30].

In the case of water extracts, each plant sample (1.0 g) was infused for 15 min with 50 mL of boiling distilled water. Then, the sample was filtered and used for further analysis as already described by Polumackanycz et al. [30].

Before high-performance liquid chromatography (HPLC) analysis, hydromethanolic and water extracts of *A. glycyphyllus* and *A. cicer* were filtered, and 20 μL of the filtrate was injected into the HPLC as already described by Polumackanycz et al. [30].

To determine tocopherols [31], approximately 500 mg of ground sample was weighed. Subsequently, 100 μL of BHT (10 mg/mL in hexane) and 400 μL of tocol (50 μg/mL) were added. Thereafter, 4 mL of methanol, 4 mL of hexane, and 2 mL of saturated aqueous NaCl solution were added, with each addition followed by vortex mixing. The mixture was then centrifuged (4000× *g* 5 min at 10 °C), and the supernatant was collected. This process was repeated three times, and the combined supernatants were evaporated to dryness under a stream of nitrogen at room temperature. Then, the samples were redissolved in HPLC-grade hexane and filtered into a vial prior to the HPLC analysis.

### 2.4. Nutritional Profile

The nutritional composition (total protein, ash, crude fats and carbohydrates) was determined in the freeze-dried samples, according to the AOAC methods [32]. The crude protein was determined by the macro-Kjeldahl method (model Pro-Nitro-A; Selecta, Barcelona, Spain) (AOAC 920.87); the fat content was determined by Soxhlet extraction with petroleum ether (AOAC 948.22); and the ash content was evaluated by incineration at 550 ± 15 °C (AOAC 923.03). Total carbohydrate content was calculated by difference using the formula: total carbohydrates (g/100 g) = 100 − (fat + ash + proteins). The energetic value was calculated using the following formula:

energy (kcal/100 g) = 4 × (gproteins + gcarbohydrates) + 9 × (gfat).

Details of the methodologies are described in Pascoalino et al. [33]. The outcomes were presented in g/100 g of dry weight (DW). The software used was the Statistical Package for the Social Sciences (SPSS) version 24 (IBM Corporation, New York, NY, USA).

### 2.5. Soluble Sugars

HPLC combined with a refraction index detector (RI) was utilized to determine the profile of soluble sugars. The HPLC consisted of an integrated system with a pump (Knauer, Smartline System1000, Berlin, Germany), an auto-sampler (AS-2057, Jasco, Easton, MD, USA), a degasser system (Smartline Manager 5000) and a refractive index detector (Knauer Smartline 2300, Berlin, Germany). The method was based on the previous procedure explained by Pascoalino et al. [33]; melezitose was utilized as an internal standard (IS). Sugars were identified and quantified comparing their retention times with authentic standard references and were expressed in g/100 g DW. The standards utilized in the analysis included sucrose, fructose, trehalose, glucose, and raffinose (Sigma-Aldrich, St. Louis, MO, USA).

### 2.6. Fatty Acids

Fatty acid methyl esters (FAME) profile was obtained after trans-esterifying the lipid fraction resulting from Soxhlet extraction. Gas–liquid chromatography combined with flame ionization detection (FID) was employed using a YOUNG IN Crhomass 6500 GC System (YL6500, Seoul, South Korea) instrument equipped with a split/splitless injector set at 250 °C, with a split ratio of 1:50. The FID was set at 260 °C, and a Zebron-Fame column (30 m × 0.25 mm ID 0.20 μm df, Phenomenex, Lisbon, Portugal) was utilized. The temperature program of the oven was as follows: preliminary temperature: 100 °C, held for 2 min; increase of 10 °C/min until reaching 140 °C; increase of 3 °C/min until reaching 190 °C; increase of 30 °C/min until reaching 260 °C. The carrier gas, hydrogen, flowed at a 1.2 mL/min rate, estimated at 250 °C. Identification and quantification were conducted by comparison of the relative retention times of FAME peaks with standards (standard mixture 47885-U, Sigma, St. Louis, USA). Results were treated with Clarity DataApex 4.0 Software (Prague, Czech Republic) and given as relative percentages.

### 2.7. Total Phenolic, Flavonoid, and Phenolic Acid Contents

Total phenolic content (TPC) was examined using the Folin-Ciocalteu method as described by Singleton and Rossi [34]. Thus, 0.2 mL of the extracts was mixed with 0.2 mL Folin-Ciocalteu (FC) reagent, and after 3 min, 2 mL of 7% sodium carbonate solution was added to the mixture and incubated in 60 min in a dark place. The absorbance values were determined at 760 nm and the results were expressed as µg of gallic acid equivalents (GAE) per g of DW.

Total flavonoid content (TFC) was determined according to the European Pharmacopoeia [35]. Then, 1 mL of each extract was mixed with 0.1 mL of 5% aluminum chloride solution and 1.4 mL of a mixture of acetic acid and methanol (19:1, *v*/*v*). After 30 min, the absorbance was measured at 425 nm and the obtained results was expressed as µg of quercetin equivalents (QE) per g of DW.

Total phenolic acid content (TPAC) was examined according to the method described in the Polish Pharmacopoeia VI [36]. Thus, the extract (1.4 mL) was mixed with hydrochloric acid (0.2 mL), Arnov’s reagent (0.2 mL), and sodium hydroxide solution (0.2 mL), as already described by Shahrivari-Baviloliaei et al. [29]. The absorbance was determined at 490 nm and the results were expressed as µg of caffeic acid equivalents (CAE) per g of DW.

TPC, TFC, TPAC, ascorbic acid (AA), and antioxidant activities were detected with a Metertech UV/Vis spectrophotometer (SP-830+, Metertech Inc., Nankang, Taipei, Taiwan) by measuring the absorbance at the appropriate wavelength described in Section 2.7, Section 2.8, and Section 2.13.

### 2.8. Determination of L(+)-Ascorbic Acid

AA was examined according to the method described by Abdelmageed et al. [37]. Then, 0.2 mL of each extract was mixed with 0.2 mL of sodium hydroxide solution, 0.2 mL of NBD-Cl solution, and 1.4 mL of 50% aqueous acetone solution. After 30 min, the absorbance was measured at 582 nm and the obtained results were expressed as µg of ascorbic acid (AA) per g of DW.

### 2.9. HPLC Analysis of Phenolic Compounds

The identification and quantification of the individual ten phenolic compounds were performed using an HPLC Merck-Hitachi LaChrome device (Darmstadt, Germany), coupled with a L-7420 UV/Vis detector. The separation was made in a Hypersil Gold C18 column (250 × 4.6 mm, 5 μm particles) (Thermo Scientific, Runcorn, UK) maintained at 30 °C according to the procedure already reported by Viapiana et al. [38]. The mobile phase consisted of 0.1% acetic acid in methanol (solvent A) and 0.1% acetic acid in water (solvent B). The gradient conditions were as follows: linear gradient from 5% to 15% of A in 10 min, from 15 to 20% in 5 min, from 20 to 30% in 5 min, from 30 to 63% in 10 min, and then isocratic elution in 5 min and a linear gradient from 63 to 5% in 5 min, and at a flow rate of 1.0 mL/min. The injection volume was 20 µL and the phenolic compounds were detected at 280 nm (GA, VA, CNA, PAT, and NAR), 320 nm (FA and *p*CA), and 370 nm (RUT, API, and Q). Phenolic compounds were identified by comparing their retention times with those of their standard compounds. Moreover, a selected sample was also spiked with the standard compounds and measured again [30].

The method used was validated in terms of linearity, the limit of detection (LOD), the limit of quantitation (LOQ), precision, and recovery, and the data are presented in Table 1. The LOQ and LOD were both determined using a signal-to-noise approach. LOQ was defined as the lowest concentration level, resulting in a peak height of 10-times the baseline noise, while LOD was calculated from the LOQ as the minimum analyte concentration with a signal-to-noise ratio equal to 3. Detailed inspection of the data indicated good linearity over the determined ranges for all the detected compounds with correlation coefficient (R^2^) values significantly higher than 0.975, while the values of LODs and LOQs were less than 4.87 μg/mL and 14.01 μg/mL, respectively. The validation parameters obtained in this study suggest excellent resolution and sensitivity of the analytical method. Moreover, the precision of the HPLC procedure met criteria for analytical methods and the coefficient of variation (CV) values were from 0.41% to 4.65% and from 0.69% to 7.64% for the intra- and inter-day variations, respectively. To assess the recovery, known quantities of the standard solutions were added to the previously analyzed extract. After extraction, a sample was processed and quantified according to the procedure described in this section. Then, the quantity of each component was subsequently calculated from the corresponding calibration curve. The mean recovery was found in the range between 90.72 and 98.11%, with a relative standard deviation (RSD) less than 2.8%. For the stability test, the retention CV was lower than 1.8% for the peak area and 0.9% for the retention time. Apart from this, the peak areas and retention times of the phenolic compounds were found to be sufficiently stable over 48 h.

### 2.10. Determination of Tocopherols

The HPLC system, referred to in Section 2.5 combined with a fluorescence detector (FP-2020; Jasco, Easton, MD, USA), was used to analyze tocopherols [39]. The detector was programmed considering an excitation wavelength of 290 nm and an emission wavelength of 330 nm. The identification was executed by comparing the chromatographic characteristics with authentic standards and further quantified (mg/100 g DW), utilizing the IS (tocol) method and calibration curves established from commercial standards (α, β, and γ-tocopherol) (Matreya, Pleasant Gap, PA, USA).

### 2.11. Mineral Profile

Flame atomic absorption technique (SpectrAA 250Plus, Varian, Australia) with an air-acetylene flame (FAAS) and a background deuterium correction was used for determination of zinc (Zn), manganese (Mn), cooper (Cu), cadmium (Cd), lead (Pb), and iron (Fe). Standard procedures were applied, and analytical wavelengths [nm] were as follows: Zn—214.0; Mn—280.0; Cu—325.0; Cd—228.8; Pb—217.0 and Fe—248.0. By comparing the absorbance responses with pure analytical solutions, the mentioned elements were detected and computed as μg/g DW.

### 2.12. Antioxidant Activity

The antioxidant activity of the *A. glycyphyllos* and *A. cicer* extracts was evaluated via three in vitro assays, i.e., DPPH, FRAP, and CUPRAC. The first method, free radical DPPH scavenging activity, was evaluated according to the protocol of Tuberoso et al. [40] with some modifications, as described by Saba Shahrivari-Baviloliaei et al. [29]. The results were expressed as mg of Trolox equivalents (TE) per g of DW [29]. FRAP assay was performed according to Benzie and Strain [41] with some modifications, as described by Saba Shahrivari-Baviloliaei et al. [29]. The results were expressed in mg of ferrous ion equivalents (Fe^2+^) per g of DW. The CUPRAC assay was implemented using the method proposed by Apak et al. [42] with some modifications, as described by Saba Shahrivari-Baviloliaei et al. [29]. The obtained results were expressed in mg ascorbic acid equivalents (AA) per g of DW.

### 2.13. Antibacterial Activity

Preliminary studies of the antimicrobial activity of *Astragalus* extracts were carried out via an agar wall diffusion test. For this study, *S. aureus* ATCC 6538 and *E. coli* ATCC 8739 strains were used according to the procedure described by Shahrivari-Baviloliaei et al. [29]. After incubation, the diameter of the zone of growth inhibition was measured. Ampicillin was used as the reference antibiotic.

### 2.14. Data Analysis

For *A. glycyphyllos* and *A. cicer*, six samples were used, and all assays were carried out in triplicate. The results are expressed as mean values and standard deviation (SD). Statistical analysis was performed using Statistica 10 software (StatSoft Inc., Tulsa, OK, USA). Moreover, one-way ANOVA was performed to study differences between analyzed samples, followed by Tukey’s honestly significant difference (HSD) test at *p* < 0.05.

## 3. Results and Discussion

### 3.1. Nutritional Profile

The nutritional profile of the different plant parts of the studied *Astragalus* species is presented in Table 2. The fruit of *A. cicer* had the highest overall total protein content, followed by leaves and roots, which showed the lowest overall content. However, in the case of *A. glycyphyllos,* the highest content was recorded in fruits, followed by leaves and roots. Regarding ash content, the highest amounts were recorded for the leaves of both species, followed by fruits, whereas roots contained the lowest amounts. Crude fat content was the highest for the roots of *A. glycypyllos*, followed by the roots of *A. cicer*, the leaves of *A. glycyphyllos* and leaves and fruits of *A. cicer*, while the fruits of *A. glycyphyllos* showed the lowest overall content. The carbohydrate content also varied among the studied species and plant parts, with fruits and roots of *A. glycyphyllos* showing the highest and lowest overall content, respectively. Finally, the highest total energy content was determined in the root of *A. glycyphyllos*, whereas the leaves of *A. cicer* had the lowest values.

Butkute et al. [43] reported similar amounts of crude protein with those of our study for the aerial parts of both *Astragalus* species, whereas the ash and carbohydrate content was higher and lower, respectively. In contrast to our study, Norman et al. [44] described that the protein content in *A. cicer* (milkvetch) was higher during the vegetative stage compared to the flowering stage, which was not the case in our study where fruits of *A. cicer* and fruits and flowers of *A. glycyphyllos* had a higher protein content compared to the leaves. Acharya et al. [45] suggested that crude protein content varied among two milkvetch cultivars, as well as between rain-fed and irrigated plants. Moreover, Foster et al. [46] suggested the significant impact of the number of harvests on protein content in milkvetch plants used for forage. Lardner et al. [47] reported a varied content of ash and crude fat between three milkvetch cultivars, while the range of the reported values differed from those of our study. This contradiction with the literature reports could be due to different growing conditions as well to differences in the genotypes tested [45,46,47]. Regarding the energy content, Norman et al. [44] indicated a high variability in *A. cicer* depending on the harvesting period, as well as on the number of harvests, thus suggesting a significant impact of the growing conditions and the agronomic practices on the nutritional value of milkvetch. Similarly, Lardner et al. [47] reported a higher energy content in three milkvetch cultivars compared to our study, which suggests a significant impact of genotype on the nutritional value of the species. To the best of our knowledge, there are scarce data regarding the nutritional value of *A. glycyphyllos,* which does not allow for any comparison of our results with those of the literature reports.

### 3.2. Free Sugars

The sugar profile varied among both the species and the plant parts (Table 3). In particular, sucrose was the most abundant in the roots of both species, especially in the case of *A. cicer,* where the highest overall content was recorded, while leaves of *A. cicer* and *A. glycyphyllos* were most abundant in fructose. However, fructose was also detected in significant amounts in roots of *A. cicer*, as well as in roots and leaves of *A. glycyphyllos.* Glucose was detected mostly in leaves of *A. cicer* and in the roots of *A. glycyphyllos*. Finally, roots of both species recorded higher amounts of total sugars compared to fruit and leaves, which contained lesser amounts. According to the literature, *A. glycyphyllos* leaves contain several monosaccharides such as pinitol, sucrose and glucose [22], while Shang et al. [48] suggested glucose and galactose as the main detected monosaccharides in the aerial parts of *A. glycyphyllos* in amounts that varied depending on the extraction method. According to Gabrielsen et al. [49], this particular pattern of carbohydrate distribution with high amounts of sugars detected in roots is due to the role of roots as energy reserves, which facilitate the regrowth of vegetation after harvesting or foraging. Moreover, Butkute et al. [43] reported a lower content of soluble sugars in the aerial parts of wild ecotypes of *A. cicer* and *A. glycyphyllos*, which indicates that growing conditions and the genotype may have an impact on the sugar composition of the species.

### 3.3. Fatty Acids

The fatty acid profile differed among the studied plant parts and the studied species (Table 4). In the case of *A. cicer*, roots were mostly abundant in linoleic and α-linolenic acid, followed by oleic, erucic and palmitoleic acid, while in fruits, oleic and palmitic acid were the richest compounds, followed by linolenic, α-linolenic and erucic acid. Leaves contained high amounts of linoleic, oleic, α-linolenic, and palmitic acid. On the other hand, the main detected fatty acids in *A.glycyphyllos* were linoleic, α-linolenicm oleic and palmitic acid in amounts that varied among the plant parts, while significant amounts of stearic and arachidic acid were also detected in fruit samples, respectively. Roots of both species showed a similar content of fatty acid groups, with PUFA and SFA being the most abundant ones. However, the rest of the plants showed a varied profile of fatty acid groups, with MUFA and PUFA being the main groups of fatty acids in *A. cicer* plant parts, whereas in *A. glycyphyllos,* SFA and/or PUFA were the richest groups of fatty acids.

In contrast to our study, Adiguzel et al. [50] detected only two fatty acids in the leaves of *A. cicer* at the flowering stage, namely behenic acid and pentacosylic acid, while the same authors suggested a great variability in the fatty acids profile among the different *Astragalus* species studied. Similar results were reported by Ağar et al. [51], who also studied the fatty acids profile in the leaves of various *Astragalus* species collected from a different region of Turkey. Moreover, Haşimi et al. [52] suggested palmitic, oleic, linoleic, α-linolenic and arachidic acid as the main fatty acids detected in the aerial parts of different *Astragalus* species, thus suggesting that these compounds are the most prevalent in the particular genus. In the study of Klichkhanov et al. [53], it was reported that the main compounds in the leaves of *A. glycyphyllos* were also α-linolenic, palmitic, linoleic and oleic acid, while 19 compounds were detected in total.

### 3.4. Total Phenolic, Flavonoid, Phenolic Acid, and L(+)-Ascorbic Acid Contents

The results obtained for TPC, TFC, TPAC, and AA, determined both in the hydromethanolic and water extracts of leaves, roots, and fruits of *A. glycyphyllos* and *A. cicer* samples, are summarized in Table 5. Generally, hydromethanolic extracts were richer in TPC, TFC, TPAC and AA than the water extracts of *A. glycyphyllos* and *A. cicer*. Many authors found methanol as the most effective solvent for phenolics extraction, but the plant material contains a wide variety of bioactive constituents [54,55,56]. Due to the variety of phytochemical compounds contained in plant materials and their differing solubility properties in different solvents, the optimal solvent for extraction also depends on the specific part of the plant material and the compounds that are to be isolated [57,58,59]. In addition to the polarity and type of the solvent and the part of the plant material, important factors in the extraction process of phenolic constituents are also the time and temperature of extraction, chemical composition, and physical characteristics of the plant materials [60]. In our study, the water extracts were prepared with the use of boiling water, and, in this case, the temperature of the water could also impact the phenolic extraction [54]. In this study, *A. cicer* extracts were significantly richer (*p* < 0.05) in phenolic compounds than *A. glycyphyllos* extracts. In hydromethanolic extracts, *A. glycyphyllos* leaves were the richest in TPC, TFC, and TPAC, while in water extracts, *A. cicer* leaves were characterized by the highest TPC, and its fruits were the richest in TFC and TPAC. Moreover, there were no significant differences between *A. glycyphyllos* and *A. cicer* fruits, excluding TPC and AA, and between their roots, excluding TPAC, TFC and AA in hydromethanolic extracts, and in water extracts between roots and fruits, excluding TPC. Comparing the results obtained in this study to those found in the literature, Myrtsi et al. [61] found TPC at the level of 16.20 mg GAE/g extract in the whole plant of *A. glycyphyllos* from Greece. Butkute et al. [62] analyzed stems, leaves, and flowers of hydroethanolic extracts of *A. glycyphyllos* and *A. cicer* and reported the highest level of TPC in leaves (26 and 17 mg GAE/g DW, for *A. glycyphyllos* and *A. cicer*, respectively), while TFC was the highest in the leaves of *A. glycyphyllos* (22 mg RE/g) and in the leaves and flowers of *A. cicer* (about 4 mg RE/g). Moreover, the stems showed the lowest values for TPC and TFC. Comparing morphological parts of other species of *Astragalus*, the hydroethanolic extracts of leaves and flowers of *A. gombiforms* were also richer in TPC and TFC than stems [63]. With regard to TPAC and AA in herbal materials from *A. glycyphyllos* and *A. cicer*, to the best of our knowledge, no published data were found.

### 3.5. Quantification of Phenolic Compounds

The quantification of the detected phenolic compounds is summarized in Table 6. The most abundant phenolic compounds in both extracts of *Astragalus* species were GA and Q. In hydromethanolic extracts, GA was detected only in *A. cicer* leaves, while FA and CNA were found only in *A. glycyphyllos* leaves and fruits, respectively. RUT was found in leaves of both *Astragalus*, while PAT, VA, pCA, API, and NAR were not detected in their extracts. In addition, there were no significant differences between *Astragalus* species fruits and roots, excluding Q in hydromethanolic extracts, and in water extracts between roots and leaves, excluding GA and Q, respectively.

These differences in phenolic compound composition compared to our study could be attributed to differences in the extraction methodology, as well as to differences in genotype and growing conditions [64,65]. To the best of our knowledge, data in the literature regarding the individual phenolic compounds in *A. glycyphyllos* are scarce. Only Myrtsi et al. [61] determined the phenolic profile in *A. glycyphyllos* and found RUT at the level of 0.16 mg/g extract, while the concentration of API and Q was not detected. In the case of *A. cicer*, to the best of our knowledge, no data of individual phenolic compounds were found in the literature.

### 3.6. Determination of Tocopherols

Tocopherols are part of the vitamin E family and occur in four homologs (e.g., α-, β-, γ-, and δ). Due to their marked antioxidant and anticancer activities, they confer health benefits, including hypolipidemic, antiatherogenic, anti-hypertensive, and neuroprotective effects [66]. The tocopherol contents *of A. glycyphylos* and *A. cicer* extracts found in the current study are exhibited in Table 7. The highest content of α-tocopherol was detected in the leaves of *A. glycyphyllos* and *A. cicer*, while the lowest amounts were detected in the roots of both species. β-Tocopherol was detected only in *A. glycyphyllos* and *A. cicer* leaves, while γ-tocopherol was present only in *A. glycyphyllos* leaves. Generally, the highest content of total tocopherols was found in the leaves of *A. glycyphyllos* and *A. cicer*, while the roots of these plants had the lowest content. The literature data on tocopherols’ content mainly refer to seed oils of *Astragalus* species because of the nutritional importance of vitamin E. For example, Bahşi et al. [67] investigated the presence of α- and γ-tocopherols in seeds of the *Astragalus* L. taxa (*A. anthlloides* Lam., *A. leporinus* var. *hirsutus*, *A. campylorhynchus*, *A. cephalotes* Banks. & Podlech var. *cephalotes*, and *A. odaratus* Lam.) and reported a varied content of α-tocopherol between 3.99 µg/g (*A. campylorhynchus*) and 11.9 µg/g (*A. cephalotes* var. *cephalotes*). Moreover, *A. anthlloides* and *A. hirsutus* had a high (over 200 µg/g) γ-tocopherol content, while in the other three species, no γ-tocopherol was detected.

### 3.7. Mineral Profile

Medicinal plants may also contain residues of toxic substances, like heavy metals, which may affect the health of humans [68]. In addition, some plants have the capacity to accumulate high concentrations of these metals without exhibiting any phytotoxic consequences [69]. It is obvious that not all metals are harmful to humans, as some of them are essential in the development of metabolic processes. For instance, Cu, Mn, Fe, and Zn are needed in small amounts for the body but become toxic after surpassing a certain concentration (1-10 ppm: part per million) [70]. Meanwhile, heavy metals like Cd and Pb are toxic to the organism, even though they can pose risks to humans through long-term exposure at very low concentrations [71].

The results obtained for element determination are presented in Table 8. In hydromethanolic extracts, Fe (2.54 μg/g DW) and Zn (2.46 μg/g DW) were at the highest levels in both *Astragalus* species, and their roots were the richest in Zn and Mn. The water extracts were also richest in Fe (2.75 μg/g DW) and Zn (2.19 μg/g DW), while *A. glycyphyllos* roots, leaves, and fruits were richer in Fe, Mn and Cu. Cd and Pb were not detected in any samples.

According to the literature, *Astragalus* species also contain other elements, for instance, copper, manganese, or cobalt, and more than 20 trace elements; however, the iron, manganese, zinc, and aluminum contents are higher than those of the others [72]. Wang et al. [73] determined the contents of K, Fe, Zn, Mn, Cu in the root and shoot of *Astragalus membranaceus* (Fisch.) and found that the content of these elements is as follows in all samples: K > Fe > Zn > Mn > Cu. However, the content of Fe, Zn and Cu in the root was richer than in the shoot. Meng et al. [74] determined Cu, Cd and Pb in roots of Chinese *Astragalus membranaceus* samples and found Cu on the same level as in this study (0.1533 mg/kg). Çaçan et al. [75] analyzed *Astragalus* taxa (*A. gummifer, A. compactus, A. lineatus* var. *longidens, A. aureus, A. onobrychis, A. declinatus, A. lineatus* var. *lineatus, A. oocephalus* subsp. *stachyophorus,* and *A. cinereus*) collected in Turkey. The authors found Fe, Mn, Cu and Zn at a higher level than in this study, 2436, 153.7, 84.5 and 14.08 mg/kg, respectively, while Cd and Pb contents were not detected in any *Astragalus* sample. Higher levels of Fe, Cu, Zn and Mn were also found in seeds of *A. meridioalis* sensu auct. from Iran, 96.54, 52.42, 120.14 and 9.076 mg/kg, respectively [76].

### 3.8. Antioxidant Activity

In this study, the antioxidant activity of *A. glycyphyllos* and *A. cicer* extracts was evaluated, and the results are shown in Table 9. Generally, *A. cicer* extracts were characterized by higher FRAP and CUPRAC assays, while in *A. glycyphyllos,* higher DPPH radical scavenging activity was found. In addition, leaves of both *Astragalus* species were characterized by higher antioxidant activities, while roots recorded the lowest DPPH, FRAP and CUPRAC values.

In the literature, Myrtsi et al. [61] found the DPPH scavenging activity in methanolic extracts of the whole *A. glycyphyllos* plant from Greece at the level of 6.50 mg TE/g extract. Butkute et al. [62] evaluated DPPH values of the hydroethanolic extracts prepared from stems, leaves and flowers of *A. glycyphyllos* from Latvia, and stems, leaves and flowers of *A. cicer* from Lithuania. In their study, the highest antioxidant activity was obtained for flowers and leaves of *A. glycyphyllos* (35.64 and 32.26 µmol/g plant material, respectively), and for leaves of *A. cicer* (128.6 µmol/g plant material). To the best of our knowledge, no data on both FRAP and CUPRAC assays of *A. cicer* and *A. glycyphyllos* extracts were found in the published literature. Regarding other species of *Astragalus*, Zengin et al. [77] analyzed extracts prepared from the aerial parts and roots of three *Astragalus* species, i.e., *A. setulosus*, *A. anthylloides*, and *A. ovalis*, and found that the higher DPPH, FRAP, and CUPRAC values were obtained for the root extract of *A. anthylloides* (44.43 mg TE/g, 99.39 mg TE/g and 144.41 mg TE/g, respectively). Lekmine et al. [63] determined the antioxidant activity with the use of DPPH assay in pods, seeds, stems, leaves and flowers of hydroethanolic extracts prepared from Algerian *A. gombiformis*, and reported that the pods recorded the highest DPPH values (306 µg/mL, IC_50_), whereas the lowest DPPH values (16.43 µg/mL, IC_50_) were obtained from flowers. Nevertheless, the results of these studies are not comparable to our work due to differences in the types of extracts and sample preparation, as well as to the expression of the results.

### 3.9. Antibacterial Activity

The antibacterial activity of water extracts from *Astragalus* infusions was tested against *S. aureus* and *E. coli* using the diffusion method on a solid medium, and the results are shown in Table 10. All the extracts of *Astragalus* species showed very similar antibacterial activity against *S. aureus* and *E. coli*. The diameter of growth inhibition zones for both bacteria ranged from 12 to 17 mm. Although plant polyphenols are generally considered to have antimicrobial activity, in this study, the antimicrobial activity of *Astragalus* water extracts suggests that a high phenolic content was not always correlated with high antibacterial activity. The same conclusion has been reached by some other researchers [78,79]. Thus, the exhibited antibacterial activity for the tested extracts could be attributed to the presence of specific phenolic compounds and the possible synergistic effects with other non-phenolic bioactive components present in the water extracts of *A. glycyphyllos* and *A. cicer*.

## 4. Conclusions

Taking into consideration the necessity to improve the knowledge regarding lesser-known *Astragalus* species, we focused this study on the nutritional, chemical, and biological activity of extracts prepared from various morphological parts of *A. glycyphyllos* and *A. cicer*. The results showed the prevalence of carbohydrates and proteins in both *Astragalus* species, particularly in their leaves and fruits. Moreover, the extracts were rich in GA and Q content, while the mineral profile showed high levels of Zn and Fe in all analyzed samples. Generally, *A. cicer* extracts presented higher phenolic contents and antioxidant activities than *A. glycyphyllos*. However, both *Astragalus* species exhibited similar antibacterial activity against *S. aureus* and *E. coli.* In conclusion, our findings support the suggestion that both *Astragalus* species may be considered as a potential source of dietary supplements and pharmaceutical and nutraceutical products, depending on species, growth stage, and plant part. However, further studies through established preclinical and clinical studies are required to confirm the therapeutic effects of *A. glycyphyllos* and *A. cicer*.

## Figures and Tables

**Table 1:** Validation parameters of the calibration curves for analytes quantified in this study (n = 3).

Analytes	Regression Equation	Linearity (μg/mL)	R^2^	LODs (μg/mL)	LOQs (μg/mL)	Recovery (%)
GA	y = 20182x − 18778	25–211	0.983	3.33	11.03	97.54
PAT	y = 37934x + 19373	23–223	0.994	3.65	9.87	95.34
CNA	y = 37373x + 13828	21–205	0.988	3.77	10.21	97.47
VA	y = 40738x + 49372	24–210	0.975	2.82	8.54	98.11
FA	y = 20389x + 15225	22–200	0.991	4.02	13.55	90.72
*p*CA	y = 96929x − 16977	23–215	0.985	4.11	14.01	93.29
API	y = 32888x + 14652	28–243	0.993	3.75	10.01	94.88
NAR	y = 45976x + 38223	26–233	0.986	3.66	9.88	92.91
RUT	y = 18779x + 26855	23–210	0.989	4.22	13.23	95.89
Q	y = 15978x + 56221	30–250	0.995	4.87	12.76	95.99

y is the peak area. x refers to the concentration of compounds (μg/mL). GA: gallic acid, PAT: protocatechuic acid, CNA: cinnamic acid, VA: vanillic acid, FA: ferulic acid, *p*CA: *p*-coumaric acid, API: apigenin, NAR: naringenin, RUT: rutin, Q: quercetin.

**Table 2:** Nutritional profile of different plant parts (roots, fruit, leaves and flowers) of the studied *Astragalus* species expressed in g/100 g DW (dried weight) and energy expressed in kcal/100 g.

Sample	Total Protein	Ash	Crude Fats	Carbohydrate Content	Total Energy
*A. glycyphyllos* L. leaves	18.00 ± 1.00 ^e^	12.00 ± 1.00 ^a^	7.00 ± 1.00 ^c^	62.00 ± 1.01 ^c^	386.00 ± 7.01 ^d^
*A. glycyphyllos* L. roots	13.02 ± 0.10 ^b^	3.50 ± 0.20 ^e^	26.00 ± 2.03 ^a^	58.00 ± 2.00 ^d^	516.03 ± 11.02 ^a^
*A. glycyphyllos* L. fruits	19.21 ± 0.10 ^d^	5.50 ± 0.30 ^c^	1.84 ± 0.03 ^e^	73.50 ± 0.32 ^a^	387.01 ± 1.00 ^d^
*A. cicer* L. leaves	20.90 ± 0.11 ^b^	13.00 ± 0.10 ^a^	3.95 ± 0.21 ^d^	62.20 ± 0.21 ^c^	367.20 ± 0.40 ^e^
*A. cicer* L. roots	16.61 ± 0.20 ^f^	4.60 ± 0.20 ^d^	15.03 ± 1.00 ^b^	63.12 ± 1.05 ^c^	459.00 ± 8.01 ^b^
*A. cicer* L. fruits	26.10 ± 0.40 ^a^	5.01 ± 0.10 ^c^	4.21 ± 0.01 ^d^	64.71 ± 0.50 ^c^	401.10 ± 0.30 ^c^

Results are presented as mean ± standard deviation. Different lowercase letters within the same column indicate significant differences among the means according to Tukey’s honestly significant difference (HSD) test at *p* < 0.05.

**Table 3:** Soluble sugars profile of different plant parts (roots, fruit, leaves and flowers) of the studied *Astragalus* species expressed in g/100 g DW.

Sample	Fructose	Glucose	Sucrose	Total Sugars
*A. glycyphyllos* L. leaves	3.80 ± 0.40 ^c^	nd	0.29 ± 0.02 ^e^	4.10 ± 0.40 ^d^
*A. glycyphyllos* L. roots	2.10 ± 0.20 ^d^	0.56 ± 0.02 ^c^	9.03 ± 1.00 ^b^	11.00 ± 1.00 ^c^
*A. glycyphyllos* L. fruits	0.42 ± 0.03 ^f^	nd	0.20 ± 0.01 ^f^	0.62 ± 0.04 ^f^
*A. cicer* L. leaves	6.40 ± 0.30 ^a^	2.30 ± 0.10 ^a^	0.90 ± 0.20 ^d^	9.70 ± 0.40 ^c^
*A. cicer* L. roots	3.00 ± 1.01 ^c^	0.30 ± 0.02 ^d^	10.48 ± 0.03 ^a^	14.10 ± 0.10 ^a^
*A. cicer* L. fruits	0.48 ± 0.01 ^e^	nd	0.92 ± 0.01 ^d^	1.40 ± 0.02 ^e^

Results are presented as mean ± standard deviation. nd—not detected. Different lowercase letters within the same column indicate significant differences among the means according to Tukey’s honestly significant difference (HSD) test at *p* < 0.05.

**Table 4:** Detailed fatty acids profile expressed of different plant parts (leaves, roots and fruits) of the studied *Astragalus* species expressed in relative percentage (%).

	*A. glycyphyllos*			*A. cicer*		
Fatty Acid	Leaves	Roots	Fruits	Leaves	Roots	Fruits
C8:0	0.44 ± 0.02 ^a^	ND	ND	ND	ND	0.12 ± 0.01 ^b^
C12:0	1.17 ± 0.01 ^a^	ND	ND	ND	ND	0.09 ± 0.10 ^b^
C14:0	ND	0.60 ± 0.03 ^d^	0.70 ± 0.10 ^c^	0.83 ± 0.02 ^b^	0.76 ± 0.00 ^c^	0.29 ± 0.02 ^e^
C15:0	ND	1.37 ± 0.05 ^a^	1.20 ± 0.01 ^b^	0.11 ± 0.01 ^e^	0.86 ± 0.03 ^c^	0.37 ± 0.01 ^d^
C16:0	14.03 ± 0.01 ^e^	26.30 ± 0.20 ^b^	34.70 ± 0.40 ^a^	9.60 ± 0.20 ^f^	25.00 ± 2.00 ^b^	12.60 ± 0.10 ^cf^
C16:1	1.34 ± 0.01 ^b^	ND	0.66 ± 0.01 ^d^	0.80 ± 0.04 ^c^	3.49 ± 0.03 ^a^	0.26 ± 0.01 ^f^
C17:0	0.49 ± 0.01 ^c^	1.27 ± 0.03 ^a^	ND	0.22 ± 0.02 ^d^	ND	0.25 ± 0.01 ^d^
C17:1	ND	ND	ND	ND	ND	0.10 ± 0.03 ^d^
C18:0	7.20 ± 0.02 ^c^	5.10 ± 0.10 ^d^	8.00 ± 0.20 ^b^	ND	4.30 ± 0.10 ^e^	ND
C18:1 n9t	ND	ND	ND	3.56 ± 0.010 ^b^	ND	4.95 ± 0.02 ^a^
C18:1 n9c	5.20 ± 0.10 ^f^	8.70 ± 0.50 ^e^	31.60 ± 0.30 ^b^	28.50 ± 0.40 ^c^	10.00 ± 1.00 ^d^	44.98 ± 0.02 ^a^
C18:2 n6c	14.30 ± 0.10 ^f^	36.90 ± 0.40 ^a^	12.00 ± 1.00 ^g^	28.70 ± 0.01 ^c^	30.00 ± 1.00 ^b^	21.1 ± 0.1 ^d^
C18:3 n3	32.22 ± 0.02 ^a^	19.80 ± 0.20 ^e^	4.70 ± 0.30 ^h^	24.80 ± 0.10 ^c^	20.00 ± 1.00 ^d^	8.8 ± 0.1 ^g^
C20:0	19.94 ± 0.02 ^a^	ND	3.41 ± 0.04 ^c^	1.1 ± 0.1 ^f^	ND	1.6 ± 0.1 ^e^
C20:1	ND	ND	ND	ND	ND	0.95 ± 0.05 ^b^
C20:3 n6	ND	ND	ND	ND	ND	0.16 ± 0.01 ^d^
C22:1 n9	3.60 ± 0.10 ^b^	ND	3.09 ± 0.04 ^c^	1.83 ± 0.04 ^e^	5.80 ± 0.10 ^a^	2.80 ± 0.10 ^d^
C23:0	ND	ND	ND	ND	ND	0.59 ± 0.04 ^b^
C24:1	ND	ND	ND	ND	ND	ND
SFA	43.27 ± 0.01 ^c^	34.60 ± 0.30 ^d^	51.00 ± 1.00 ^a^	11.80 ± 0.40 ^g^	31.00 ± 2.00 ^e^	15.95 ± 0.01 ^f^
MUFA	10.20 ± 0.10 ^e^	8.71 ± 0.50 ^f^	32.20 ± 0.30 ^c^	34.70 ± 0.30 ^b^	19.00 ± 1.00 ^d^	54.00 ± 0.20 ^a^
PUFA	46.60 ± 0.10 ^d^	56.71 ± 0.20 ^a^	17.00 ± 1.00 ^g^	53.50 ± 0.10 ^b^	50.00 ± 1.00 ^c^	30.00 ± 0.20 ^f^

ND—not detected. Results are presented as mean ± standard deviation. C8:0—Caprylic acid; C12:0—Lauric acid; C14:0—Myristic acid; C15:0—Pentadecanoic acid; C16:0—Palmitic acid; C16:1—Palmitoleic acid; C17:0—Heptadecanoic acid; C17:1—Heptadecenoic acid; C18:0—Stearic acid; C18:1n9t and C18:1n9c—Oleic acid (trans and cis); C18:2n6c—Linoleic acid; C18:3n3—α-Linolenic acid; C20:0—Arachidic acid; C20:1—Eicosenoic acid; C20:3n6—Dihomo-gamma-linolenic acid; C22:1n9—Erucic acid; C23:0—Tricosylic acid; C24:1—nervonic acid. SFA—saturated fatty acids; MUFA—monounsaturated fatty acids; PUFA—polyunsaturated fatty acids. Different lowercase letters within the same row indicate significant differences among the means according to Tukey’s honestly significant difference (HSD) test at *p* < 0.05.

**Table 5:** Total phenolic (TPC), total phenolic acid (TPAC), total flavonoid (TFC), and ascorbic acid (AA) contents in the hydromethanolic and water extracts of different parts of *A. glycyphyllos* and *A. cicer*.

Sample	TPC (μg GAE/g DW)	TPAC (μg CAE/g DW)	TFC (μg QE/g DW)	AA (μg AA/g DW)
Hydromethanolic extracts				
*A. glycyphyllos* leaves	6.41 ± 1.42 ^c^	4.35 ± 0.20 ^b^	2.43 ± 0.22 ^c^	2.08 ± 0.44 ^a^
*A. glycyphyllos* roots	0.88 ± 0.67 ^a^	0.54 ± 0.08 ^a^	0.12 ± 0.02 ^a^	2.27 ± 0.51 ^a^
*A. glycyphyllos* fruits	1.60 ± 0.44 ^a^	0.34 ± 0.02 ^a^	0.76 ± 0.08 ^a^	1.77 ± 0.15 ^a^
*A. cicer* leaves	8.30 ± 0.24 ^d^	5.62 ± 0.25 ^c^	3.27 ± 0.65 ^b^	4.14 ± 0.16 ^b^
*A. cicer* roots	3.67 ± 0.91 ^b^	0.52 ± 0.07 ^a^	0.21 ± 0.05 ^a^	1.93 ± 0.05 ^a^
*A. cicer* fruits	1.64 ± 0.50 ^a^	6.93 ± 0.78 ^d^	3.26 ± 0.37 ^b^	2.22 ± 0.27 ^a^
Water extracts				
*A. glycyphyllos* leaves	70.83 ± 1.82 ^c^	355.42 ± 4.54 ^b^	1172.62 ± 18.49 ^a^	13.56 ± 4.85 ^a^
*A. glycyphyllos* roots	15.33 ± 0.24 ^a^	1280.82 ± 14.33 ^e^	1183.14 ± 16.43 ^a^	9.54 ± 0.06 ^b^
*A. glycyphyllos* fruits	27.31 ± 0.23 ^b^	637.22 ± 2.29 ^f^	788.19 ± 6.10 ^b^	9.25 ± 0.16 ^b^
*A. cicer* leaves	78.29 ± 2.03 ^d^	285.07 ± 8.81 ^a^	1104.65 ± 13.12 ^d^	12.81 ± 2.03 ^a^
*A. cicer* roots	13.42 ± 0.48 ^a^	878.33 ± 9.19 ^d^	953.95 ± 12.94 ^c^	23.39 ± 1.41 ^c^
*A. cicer* fruits	24.53 ± 0.41 ^b^	1702.16 ± 17.79 ^c^	1140.87 ± 14.64 ^e^	12.13 ± 0.92 ^a^

The results in the same column followed by the same letters do not significantly differ by Tukey’s HSD test (*p* < 0.05).

**Table 6:** The content of phenolic compounds (μg/g DW) of hydromethanolic and water extracts from different parts of *A. glycyphyllos* and *A. cicer* (mean ± SD).

Sample	GA	PAT	VA	*p*CA	FA	CNA	API	NAR	RUT	Q
Hydromethanolic extracts										
*A. glycyphyllos* leaves	89.93 ± 0.84 ^a^	ND	ND	ND	13.83 ± 1.64	ND	ND	ND	2.79 ± 2.64 ^a^	336.27 ± 6.24 ^d^
*A. glycyphyllos* roots	75.82 ± 1.33 ^a^	ND	ND	ND	ND	ND	ND	ND	ND	35.62 ± 1.76 ^a^
*A. glycyphyllos* fruits	98.22 ± 1.69 ^a^	ND	ND	ND	ND	2.34 ± 1.83	ND	ND	72.17 ± 1.37 ^b^	48.00 ± 2.40 ^b^
*A. cicer* leaves	49.84 ± 6.55 ^a^	ND	ND	ND	ND	ND	ND	ND	4.53 ± 1.56 ^a^	82.78 ± 2.11 ^c^
*A. cicer* roots	ND	ND	ND	ND	ND	ND	ND	ND	ND	37.27 ± 3.46 ^a^
*A. cicer* fruits	ND	ND	ND	ND	ND	ND	ND	ND	ND	51.15 ± 5.18 ^b^
Water extracts										
*A. glycyphyllos* leaves	355.43 ±8.76 ^c^	ND		ND	82.65 ± 5.54	ND	ND	ND	308.93 ± 6.81	45.99 ± 0.52 ^a^
*A. glycyphyllos* roots	302.15 ±7.37 ^a^	ND	ND	ND	ND	ND	ND	ND	ND	43.71 ± 0.12 ^a^
*A. glycyphyllos* fruits	674.50 ± 12.22 ^e^	ND	ND	ND	ND	6.17 ± 2.82	ND	ND	ND	264.15 ± 10.82 ^d^
*A. cicer* leaves	435.49 ± 13.75 ^d^	ND	ND	ND	ND	ND	ND	ND	ND	45.80 ± 0.30 ^a^
*A. cicer* roots	307.79 ± 7.31 ^a^	ND	ND	ND	ND	ND	ND	ND	ND	61.78 ± 1.41 ^b^
*A. cicer* fruits	347.40 ± 1.18 ^b^	ND	ND	ND	ND	ND	ND	ND	ND	75.60 ± 1.02 ^c^

The results in the same column followed by the same letters do not significantly differ by Tukey’s HSD test (*p* < 0.05); ND—not detected; GA—gallic acid, PAT—protocatechuic acid, CNA—cinnamic acid, VA—vanillic acid, FA—ferulic acid, *p*CA—*p*-coumaric acid, API—apigenin, NAR—naringenin, RUT—rutin, Q—quercetin.

**Table 7:** Composition of tocopherols of the studied different parts of *A. glycyphyllos* and *A. cicer* (g/100 g DW).

Sample	α-Tocopherol	β-Tocopherol	γ-Tocopherol	Total
*A. glycyphyllos* leaves	88 ± 4 ^a^	2.7 ± 0.5 ^a^	1.0 ± 0.1	92 ± 4 ^a^
*A. glycyphyllos* roots	0.20 ± 0.04 ^e^	ND	ND	0.20 ± 0.04 ^e^
*A. glycyphyllos* fruits	2.7 ± 0.5 ^d^	ND	ND	2.7 ± 0.5 ^d^
*A. cicer* leaves	40 ± 6 ^b^	0.6943 ± 0.0002 ^b^	ND	40 ± 6 ^b^
*A. cicer* roots	0.173 ± 0.001 ^e^	ND	ND	0.173 ± 0.001 ^e^
*A. cicer* fruits	9 ± 1 ^c^	ND	ND	9 ± 1 ^c^

Results are presented as mean ± SD (n = 3), ND—not detected. The results in the same column followed by the same letters do not significantly differ by Tukey’s HSD test (*p* < 0.05).

**Table 8:** The content of Pb, Fe, Cd, Cu, Zn and Mn (μg/g DW) in hydromethanolic and water extracts prepared from different parts of *A. glycyphyllos* and *A. cicer*.

Sample	Pb	Fe	Cd	Cu	Zn	Mn
Hydromethanolic extracts						
*A. glycyphyllos* leaves	ND	2.66 ± 0.23 ^b^	ND	0.73 ± 0.09 ^c^	1.77 ± 0.23 ^a^	0.65 ± 0.09 ^b^
*A. glycyphyllos* roots	ND	2.87 ± 0.64 ^c^	ND	0.52 ± 0.09 ^ab^	3.22 ± 0.04 ^c^	1.28 ± 0.45 ^cd^
*A. glycyphyllos* fruits	ND	3.44 ± 0.88 ^d^	ND	0.49 ± 0.07 ^a^	2.19 ± 0.17 ^b^	0.95 ± 0.23 ^c^
*A. cicer* leaves	ND	2.01 ± 0.56 ^b^	ND	0.43 ± 0.10 ^a^	3.43 ± 0.48 ^c^	0.93 ± 0.76 ^c^
*A. cicer* roots	ND	2.42 ± 0.87 ^b^	ND	0.62 ± 0.04 ^b^	1.81 ± 0.09 ^a^	0.54 ± 0.08 ^a^
*A. cicer* fruits	ND	1.83 ± 0.76 ^a^	ND	0.75 ± 0.08 ^c^	2.35 ± 0.10 ^b^	0.66 ± 0.05 ^b^
Water extracts						
*A. glycyphyllos* leaves	ND	4.1 ± 0.13 ^c^	ND	2.91 ± 0.22 ^d^	3.1 ± 0.23 ^d^	1.93 ± 0.16 ^d^
*A. glycyphyllos* roots	ND	2.5 ± 0.13 ^b^	ND	0.38 ± 0.56 ^a^	1.5 ± 0.35 ^a^	0.63± 0.06 ^bc^
*A. glycyphyllos* fruits	ND	4.0 ± 0.29 ^c^	ND	0.42 ± 0.32 ^ab^	2.4 ± 0.11 ^c^	0.54 ± 0.02 ^b^
*A. cicer* leaves	ND	2.32 ± 0.10 ^b^	ND	0.48 ± 0.34 ^ab^	2.5 ± 0.21 ^c^	0.65 ± 0.09 ^bc^
*A. cicer* roots	ND	1.72 ± 0.29 ^a^	ND	0.65 ± 0.21 ^b^	1.7 ± 0.15 ^ab^	0.71 ± 0.05 ^c^
*A. cicer* fruits	ND	1.85 ± 0.16 ^a^	ND	1.33 ± 0.33 ^c^	1.9 ± 0.13 ^b^	0.34 ± 0.02 ^a^

The results in the same column followed by the same letters do not significantly differ by Tukey’s HSD test (*p* < 0.05). ND—not detected.

**Table 9:** Antioxidant activities of *A. glycyphyllos* and *A. cicer* extracts.

Sample	DPPH (mg TE/g DW)	FRAP (mg Fe^2+^/g DW)	CUPRAC (mg AA/g DW)
Hydromethanolic extracts			
*A. glycyphyllos* leaves	4.54 ± 0.18 ^a^	130.60 ± 8.38 ^e^	34.77 ± 1.99 ^d^
*A. glycyphyllos* roots	1.99 ± 0.72 ^b^	47.77 ± 5.51 ^d^	3.43 ± 0.62 ^a^
*A. glycyphyllos* fruits	5.02 ± 0.25 ^a^	34.15 ± 1.25 ^b^	19.17 ± 1.72 ^b^
*A. cicer* leaves	4.54 ± 0.34 ^a^	290.21 ± 12.25 ^f^	160.60 ± 7.59 ^e^
*A. cicer* roots	2.17 ± 1.07 ^b^	26.56 ± 3.78 ^a^	2.92 ± 0.28 ^a^
*A. cicer* fruits	4.76 ± 0.22 ^a^	42.38 ± 2.94 ^c^	23.20 ± 1.67 ^c^
Water extracts			
*A. glycyphyllos* leaves	123.58 ± 5.11 ^d^	44.39 ± 1.38 ^c^	12.48 ± 1.02 ^c^
*A. glycyphyllos* roots	52.15 ± 7.61 ^b^	10.36 ± 0.56 ^b^	1.65 ± 0.21 ^a^
*A. glycyphyllos* fruits	111.73 ± 3.03 ^c^	5.49 ± 0.80 ^a^	2.08 ± 0.70 ^a^
*A. cicer* leaves	0.184 ± 0.02 ^a^	52.66 ± 0.91 ^d^	45.12 ± 1.11 ^d^
*A. cicer* roots	0.047 ± 0.00 ^a^	5.68 ± 0.17 ^a^	2.11 ± 0.8 ^a^
*A. cicer* fruits	0.115 ± 0.01 ^a^	8.96 ± 0.46 ^b^	6.56 ± 1.35 ^b^

The results in the same column followed by the same letters do not significantly differ by Tukey’s HSD test (*p* < 0.05).

**Table 10:** Antibacterial activity of water extracts from different parts of *A. glycyphyllos* and *A. cicer*.

Samples	*S. aureus* ATCC6538	*E. coli* ATCC8739
300 mg/L, 300 μL		
*A. glycyphyllos* leaves	16	12
*A. glycyphyllos* roots	12	17
*A. glycyphyllos* fruits	13	17/12
*A. cicer* leaves	16	15
*A. cicer* roots	12/13	16
*A. cicer* fruits	15/15	12
Ampicillin—control/reference		
2 mg	44	31
0.2 mg	35	24
0.02 mg	27	15
0.002 mg	18	nz

## Data Availability

The original contributions presented in this study are included in the article. Further inquiries can be directed to the corresponding authors.
